# Brain iron accumulation in unexplained fetal and infant death victims with smoker mothers-The possible involvement of maternal methemoglobinemia

**DOI:** 10.1186/1471-2431-11-62

**Published:** 2011-07-06

**Authors:** Anna M Lavezzi, Lucijan Mohorovic, Graziella Alfonsi, Melissa F Corna, Luigi Matturri

**Affiliations:** 1 "Lino Rossi" Research Center for The Study and Prevention of Unexpected Perinatal Death and SIDS, Department of Surgical, Reconstructive and Diagnostic Sciences, University of Milan, Italy; 2 Department of Environmental Medicine, University of Rijeka School of Medicine, Rijeka, Croatia

**Keywords:** SIUD, SIDS, brain iron injury, oxidative stress, methemoglobin

## Abstract

**Background:**

Iron is involved in important vital functions as an essential component of the oxygen-transporting heme mechanism. In this study we aimed to evaluate whether oxidative metabolites from maternal cigarette smoke could affect iron homeostasis in the brain of victims of sudden unexplained fetal and infant death, maybe through the induction of maternal hemoglobin damage, such as in case of methemoglobinemia.

**Methods:**

Histochemical investigations by Prussian blue reaction were made on brain nonheme ferric iron deposits, gaining detailed data on their localization in the brainstem and cerebellum of victims of sudden death and controls. The Gless and Marsland's modification of Bielschowsky's was used to identify neuronal cell bodies and neurofilaments.

**Results:**

Our approach highlighted accumulations of blue granulations, indicative of iron positive reactions, in the brainstem and cerebellum of 33% of victims of sudden death and in none of the control group. The modified Bielschowsky's method confirmed that the cells with iron accumulations were neuronal cells.

**Conclusions:**

We propose that the free iron deposition in the brain of sudden fetal and infant death victims could be a catabolic product of maternal methemoglobinemia, a biomarker of oxidative stress likely due to nicotine absorption.

## Background

In mammals, iron is a vital constituent of the oxygen-carrrier hemoglobin (Hb). Human Hb is a tetramer consisting of a pair of α-like globin chains and a pair of β-like chains. Each chain is bound to a prosthetic heme group, consisting of an iron atom in the ferrous state located at the center of a porphyrin ring; this structure has a high affinity for oxygen. Thus, Hb is best known for its oxygen-carrying capacity, which facilitates the transport of oxygen from the lungs to the tissues [1-3].

There are numerous causes of hemoglobin-related diseases. A distinction can be made between genetically inherited diseases, such as thalassemias and sickle cell disease [4-6], and acquired disorders, such as methemoglobinemia [7-9], a rare condition characterized by the presence of a greater concentration than the normal physiological range of 1-2% methemoglobin in erythrocytes.

Despite their different causes, these Hb disorders all arise from an oxidative denaturation of Hb. Oxidative injury can give rise to hemolysis with the consequent release into the circulation of Hb denatured products and of ferric iron (Fe^3+^), that is the oxidised form of the normal reduced ferrous (Fe^2+^) state. This free iron is characterized by its inability to bind and transport oxygen [10-12]. During pregnancy, it can cross the placental-fetal barrier and inhibit the release of oxygen into fetal tissues, inducing hypoxia. Ferric iron also readily permeates through the fetal blood-brain barrier, causing, besides brain hypoxia, direct long-term DNA damage to neuronal cells regarded as selectively vulnerable [13].

We previously reported a high susceptibility of the autonomic nervous system (ANS) to oxidative stress caused by maternal cigarette smoking in pregnancy, with consequent alterations of nuclei and/or structures controlling the vital activities, in victims of sudden fetal and infant death [14-17]. In this study, we aimed to evaluate whether oxidative metabolites of nicotine could also affect iron homeostasis in the brain of these victims, maybe through the induction of maternal Hb damage. The study was conducted by investigating, by means of Perls' Prussian Blue reaction, the possible presence of free iron in the ferric form in brain tissues of victims of unexplained death, aged from 25 gestational weeks to 10 postnatal months, and evaluating a possible relation with maternal smoking in pregnancy.

## Methods

In total, 56 brains were collected from 24 fresh stillbirths (25-40 gestational weeks, with a peak from 36 to 40 weeks) and 32 infants aged 1-10 months (mean age: 3 months).

This was a selected set of cases, all sent to our Research Center in application of the 2006 guidelines stipulated by Italian law n.31 **"**Regulations for Diagnostic Post Mortem Investigation in Victims of sudden infant death syndrome (SIDS) and sudden intrauterine unexpected death (SIUD)**"**. This law decrees that all infants with suspected SIDS who died suddenly in Italian regions within the first year of age, as well as all fetuses who died after the 25^th ^week of gestation without any apparent cause, must undergo an in-depth anatomo-pathological examination, particularly of the autonomic nervous system [18,19].

Ethics-Ethics approval for this study was granted by the Italian Health's Ministry in accordance with the above-mentioned Italian Law n. 31. Parents of all subjects (SIDS, SIUD) and controls provided written informed consent to both autopsy and genetic study, under protocols approved by the Milan University L. Rossi Research Center institutional review board.

After fixation in 10% phosphate-buffered formalin, the brainstem and cerebellum, the main structures analyzed in our studies, were processed and embedded in paraffin.

Transverse serial sections of the midbrain, pons, medulla oblongata, and cerebellum samples were made at intervals of 50-60 μm. For each level, serial 5 μm sections were obtained, two of which were routinely stained for histological examination using hematoxylin-eosin and Klüver-Barrera and one was submitted to Perls' Prussian Blue reaction for histochemical demonstration of the ferric iron content. In addition, in selected cases (showing brain iron accumulation by Perls' Prussian Blue reaction), adjacent sections were submitted to a modified Bielschowsky's method (Gless and Marsland's modification). This is the method of choice in the writer's experience for visualizing nervous cells, and thus evaluating if the cells with iron granulations are really neurons. The remaining sections were saved and stained as deemed necessary for further investigations.

In the Perls' Prussian Blue reaction [20] the ferric iron, released from an attachment to protein by treatment with diluted hydrochloric acid, reacts with a diluted solution of potassium ferrocyanide to produce an insoluble blue compound, namely ferric ferrocyanide (Prussian Blue), according to the following reaction:

Practically, sections were firstly deparaffinized and hydrated in distilled water, then immersed for 60 minutes in a mix of equal parts of hydrochloric acid 2% in aqueous solution and potassium ferrocyanide 2% of aqueous solution prepared immediately before use. The slides were afterwards washed in distilled water and counterstained with Kernechtrot solution (0.1 gr of nuclear fast red + 5 g of aluminum sulfate dissolved in 100 ml of distilled water, followed by filtration and addition of thymol as preservative).

The Gless and Marsland's modification of Bielschowsky's method is a development of the silver impregnation method that basically depends on silver oxide (or hydroxide) dissolved in ammoniacal solution. This method selectively allows to a brown stain of neuronal cell bodies and neurofilaments [21].

The routine histological evaluation of the brainstem was focused on the locus coeruleus and the parabrachial/Kölliker-Fuse complex in the rostral pons/caudal mesencephalon; on the retrotrapezoid nucleus, the superior olivary complex and the facial/parafacial complex in the caudal pons; on the hypoglossus, the dorsal motor vagal, the tractus solitarius, the ambiguus, the pre-Bötzinger, the inferior olivary, the raphé and the arcuate nuclei in the medulla oblongata.

In the cerebellum, the cortex layers (external granular layer, molecular layer, Purkinje cell layer and internal granular layer) and the medullary deep nuclei (the dentate nucleus, the fastigial nucleus, the globose nucleus and the emboliform nucleus) were examined.

In 36 cases, after the in-depth histopathological examination the death remained totally unexplained. A diagnosis of sudden intrauterine unexplained death (SIUD) was established for 16 fetuses, who died suddenly after the 25^th ^gestational week before complete expulsion or retraction from the mother. Sudden infant death syndrome (SIDS) was diagnosed in 20 infants who died within the first year of life. In the remaining 20 cases, 9 stillbirths, and 11 infant deaths, a precise cause of death was formulated at autopsy. These cases were used as controls. The related infant death diagnoses in this group were: congenital heart disease (n = 5), severe bronchopneumonia (n = 2), myocarditis (n = 1), pulmonary dysplasia (n = 2), and mucopolysaccharidosis type I (n = 1). Specific diagnoses among the fetal deaths included: chorioamnionitis (n = 6) and congenital heart disease (n = 3).

For every case, a complete clinical history was collected. Additionally, mothers were asked to complete a questionnaire on their smoking habit, detailing the number of cigarettes smoked before, during and after pregnancy. Fifteen of the 36 SIDS/SIUD mothers (42%) were active smokers before and during the pregnancy, smoking more than 3 cigarettes/day. The remaining 21 mothers (58%) admitted no history of cigarette smoking. Four of the 20 mothers in the control group (20%) reported a smoking habit, while the remaining 16 mothers (80%) were non smokers.

### Statistical analysis

Spearman's rho rank correlation test was used to test the correlation between maternal smoking and alterations of brain iron homeostasis in the groups of victims. Statistical calculations were carried out on a personal computer with SPSS statistical software (version 11.0; SPSS Inc., Chicago, IL, USA). The selected threshold level for statistical significance was p < 0.05.

## Results

**Figure 1:**
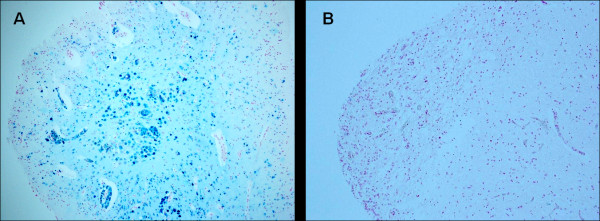
**Area postrema- **A) Ferric iron positivity in a SIUD victim who died at 36 gestational weeks (case no.3). B) Free iron negativity in an age-matched control case. Staining: Perls' Prussian Blue reaction for ferric iron-Magnification A) B): 10×.

**Figure 2:**
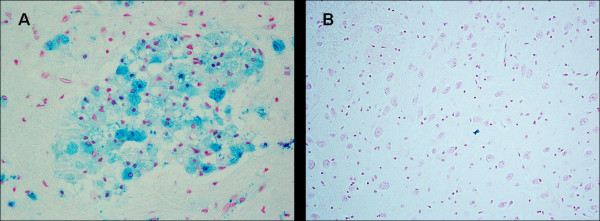
**Pontine basal nuclei-** A) Ferric iron positivity in a SIUD victim who died at 32 gestational weeks (case no.4). B) Free iron negativity in an age-matched control case. Staining: Perls' Prussian Blue reaction for ferric iron-Magnification A) B): 20×.

**Figure 3:**
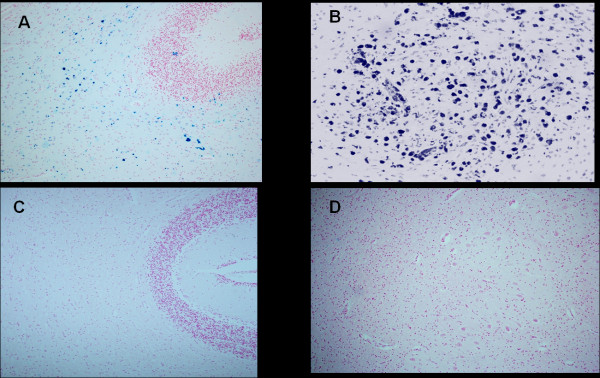
**Cerebellum-Ferric iron positivity in**: A) subcortical region and B) parenchyma in the dentate nucleus area in a 6 month-old victim of SIDS (case no.9). In C) and D) equivalent iron-negative regions in an age-matched control case. Staining: Perls' Prussian Blue reaction for ferric iron-Magnification A)C): 10x; B)D): 20×.

**Figure 4:**
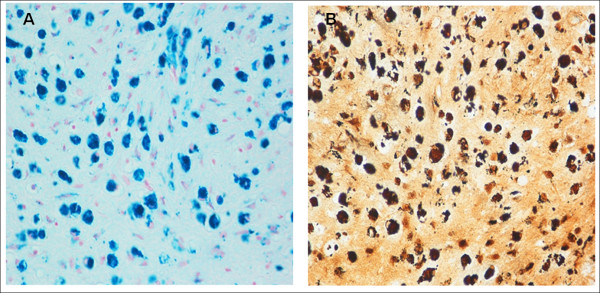
**Comparison between Perls' Prussian Blue reaction (in A) and modified Bielschowsky's method (in B) in consecutive brain histological sections of a 7 month-old SIDS case (no.8)**. The cells with blue iron accumulations in A correspond to brown neuronal cells in B-Magnification A)B): 20×.

**Figure 5:**
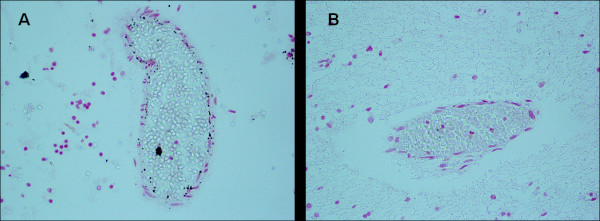
**Blood-brain barrier vessels- **A) Iron positivity in endothelial cells of a victim of SIUD aged 36 gestational weeks (case no.3). B) Iron negativity in an age-matched control case. Staining: Perls' Prussian Blue reaction for ferric iron-Magnification A) B): 20×.

Application of the Blue Prussian method highlighted accumulations of blue granulations, indicative of nonheme Fe^3+^-positive reactions, in the brainstem and cerebellum of 12 (33%) of the 36 SIUD/SIDS victims and in none of the control group. In the positive cases, iron deposits were widespread in brain parenchyma or localized in specific areas showing a variable extent and intensity. We observed large areas with a strong deposition of blue granules, particularly in the area postrema (Figure 1), in the basal nuclei of the pons (Figure 2) and in cerebellar parenchyma, both in the subcortical region, below the internal granular layer, and in the area of the dentate nucleus (Figure 3). A less marked positivity was seen in the field of the trigeminal nucleus. The iron deposits were scattered along the interstitium or, more frequently, concentrated in the neuronal cytoplasma. In these cases, the modified Bielschowsky's method confirmed that the cells with iron accumulations were neuronal cells (Figure 4). The observation of mitotic or multinucleated iron-positive neurons was not uncommon. Besides, we visualized an iron positive reaction in endothelial cells of many capillaries in the blood-brain barrier (Figure 5).

Table 1 shows the regional distribution of Fe^3+ ^in the brains of the 12 victims of sudden death showing an iron positive reaction, as well as correlations of the iron findings with both morphological alterations and a maternal smoking habit.

**Table 1:** Regional distribution of Fe^3+ ^in brain of SIUD/SIDS victims and correlation with the alterations of the central nervous system and with the maternal smoking habit

**Case no**.	Diagnosis	Age at death	BBB	Iron positivity				CNS structural alterations	Maternal smoking
				Brainstem	localization	Cerebellum	localization		Yes
1	SIUD	39 gw	+	++ +	inferior olivary n trigeminal n.	+	dentate n.	arcuate n. hypoplasia pre-Bötzinger n. hypoplasia parafacial n. hypoplasia	No

2	SIUD	41 gw	-	+	diffuse	++	subcortical	parafacial n. hypoplasia	Yes

3	SIUD	36 gw	+++ -	+++ +	area postrema tractus solitarius n	-	-	parafacial n. hypoplasia	Yes

4	SIUD	32 gw	+	++	diffuse	-	-	arcuate n. hypoplasia raphè n. hypoplasia	Yes

5	SIUD	40 gw	++	+	superior olivary n	++ +++	Subcortical dentate n.	parabrachial/Köllike-rFuse hypoplasia	Yes

6	SIDS	4 m	++	++	area postrema*	++	dentate n.	cerebellar cortex immaturity	Yes

7	SIDS	3 m	-	+++	basal pontine n*	++	diffuse	pre-Bötzinger n. hypoplasia	Yes

8	SIDS	7 m	++	+++	basal pontine n*	+++ +++	subcortical dentate n.	arcuate n. hypoplasia raphè n. hypoplasia inferior olivary n. hypoplasia	Yes

9	SIDS	6 m	++	++	diffuse*	+++ +++	subcortical dentate n.	raphè n. hypoplasia	Yes

10	SIDS	2 m	-	++	diffuse	++	diffuse	raphè n. hypoplasia cerebellar cortex immaturity	Yes

11	SIDS	3 m	++	++	diffuse	+	diffuse	arcuate n. hypoplasia raphè n. hypoplasia	Yes

12	SIDS	1 m	-	+ ++	trigeminal n. diffuse	++ ++	subcortical dentate n.	hypoglossal n. hypoplasia raphè n. hypoplasia	Yes

The intensities of iron deposition were: weak (+), strong (++), very strong (+++)

gw = gestational weeks; m = postnatal months

BBB = blood/brain barrier; n = nucleus

* = presence of mitotic and/or multinucleated neurons.

A highly significant correlation was evident between maternal smoking and alterations of brain iron homeostasis. In fact, in 11 of the 12 victims the mother smoked before and during pregnancy (p < 0.01).

In addition, routine histological examination of the autonomic nervous system showed structural alterations of various brainstem and cerebellum structures. Frequently we observed hypoplasia/agenesis of the arcuate nucleus, pre-Bötzinger nucleus, inferior olivary nucleus, serotonergic raphé nuclei, parabrachial/Kölliker-Fuse nucleus, parafacial nucleus in the medulla oblongata/pons, and delayed cerebellar cortex maturation.

## Discussion

Iron is indispensable for vital biological functions such as oxygen transport, mitochondrial energy production, DNA synthesis and DNA repair [22-24]. At least 75% of the body's iron is found within erythrocytes in the form of "heme iron". "Nonheme" iron is bound prevalently to transferrin, an abundant circulating protein that binds and delivers iron throughout the systemic circulation to all tissues, except the central nervous system (CNS) that is separated from the systemic circulation by the blood-brain barrier (BBB) [25].

Crossing of the iron-transferrin complex to the brain occurs by receptor-mediated endocytosis through endothelial cells of BBB capillaries [26-28]. This complex can then circulate in the brain interstitium, supplying iron to neuronal cells and participating in important neuronal functions, thanks to its ability to serve as both an electron donor and acceptor. In particular, its contribution to neurotransmitter synthesis, myelin formation and other crucial vital processes such as DNA synthesis makes iron essential for normal development and functioning of the CNS [29,30].

Moreover, the same chemical properties that make bound iron a highly versatile component in numerous vital activities can cause toxicity if the iron is unshielded. Iron toxicity is largely based on "Fenton chemistry", whereby excess free iron reacts with reactive oxygen intermediates, including hydrogen peroxide (H_2_O_2_) and the superoxide anion (O_2_^-^)-both by-products of aerobic metabolism-to produce an increase in reactive oxygen species (ROS) such as the hydroxyl radical (OH^·^) [31].

ROS are highly unstable due to the presence of unpaired electrons that are responsible for initiating oxidation. Even if, under normal conditions, free radical species are important for biological functions such as fighting infection or coordinating inflammation [32], excessive free radicals production can induce tissue damage by attacking lipids, DNA, and proteins [33,34].

In this study we investigated the presence of free iron accumulations in brain histological sections in a wide set of victims of sudden fetal death and SIDS, making use of Perl's Prussian Blue reaction. In this, one of the oldest histochemical methods ferric iron, if present, reacts with a dilute solution of potassium ferrocyanide to produce an insoluble blue compound (Prussian blue).

We observed diffuse blue granulations, scattered and/or assembled in different brainstem and cerebellum areas, in a consistent subset of these victims (33%) but in none of the control cases. Of particular interest was the finding, in 4 SIUD and 4 SIDS victims, of a positive iron reaction in endothelial cells of capillaries of the blood-brain barrier. Capillaries in the CNS are unusual in that they are sealed with tight junctions, without the fenestrations that characterize capillaries of the systemic circulation. So, drugs and nutrients cannot pass between these cells. Furthermore, the oxidised free iron is promptly seized by the transferrin receptors that densely populate the endothelial cells of BBB capillaries [27,35], to immobilize it and so to protect neuronal cells from oxidative damage. In fact, brain cells have relatively low antioxidant defenses [36] such as superoxide dismutase, catalase, and glutathione, that usually work in conjunction to combat free radicals [37].

Despite this defensive mechanism, experimental studies have demonstrated that non-transferrin-bound iron is able to readily cross the BBB, at a faster rate of transfer than the complex formation [27,38]. The observation, in our 8 cases, that the positive reaction in BBB capillary endothelial cells was constantly associated to widespread iron deposits in different neuronal regions, confirms these studies.

The finding in 4 SIDS victims of mitotic figures and bi/multinucleated iron-positive neurons was noteworthy. It is postulated that in prenatal CNS ontogeny, neuroblasts from the neuroepithelial matrix undergo mitotic activity and migrate to reach their definitive location, where they differentiate into mature neurons [39]. So, after birth, the mitotic activity has ceased in fully differentiated neurons.

We believe that the nuclear divisions we observed in this study are a mitotic response to the presence of a cytoplasmic iron excess, in agreement with different studies suggesting a role of intracellular iron in the induction of replication activities. In particular, Anderson et al. demonstrated that iron can interfere with the p21 gene, which is involved in the activation of genes related to DNA replication and mitosis [40]. In a study of human prostatic and renal cancers, Nemoto et al. [41] claimed that iron cooperates with zinc to activate telomerase, and by maintaining a sufficient chromosomal telomere length to ensure DNA replication and an unlimited number of irregular cell divisions.

The induction of both DNA synthesis and nuclear division in mature nervous cells of SIDS victims could imply the formation of daughter nuclei with an imbalanced DNA content, resulting in uncontrolled neuronal transformation and in disruption of the CNS homeostasis.

In addition, we propose a correlation between the widespread presence of iron molecules and the structural alterations observed in different nuclei and/or structures of the brainstem and cerebellum. In fact, free iron may induce neuronal damage by directly acting on DNA, proteins and other cellular components through an excessive production of reactive free radicals [42]. In support of this hyopothesis, different works have shown that iron accumulation in the brain contributes to neurodegenerative diseases, notably Alzheimer's disease, Parkinson's disease and Huntington's disease [43-45].

We also envisage the possibility that Prussian blue-positive reactions indicate accumulations of "insoluble" ferric iron molecules that are unavailable for cellular use, resulting in a neuronal iron deficiency. Given the importance of high iron uptake in brain development [29,30], this imbalance could contribute to the pathogenic mechanisms underlying sudden fetal death and SIDS.

The mechanisms that lead to iron accumulation are not completely understood.

Iron accumulations observed in the brain of fetuses and infants could be ascribed to an immaturity of the regulatory system of the iron homeostasis, that is particularly critical during periods of rapid growth and differentiation such as the fetal and neonatal stages [46]. Another plausible explanation could be the diffusion of oxidants into the circulation. Neonatal and fetal hemoglobin in these conditions are more likely to release free iron than adult hemoglobin [47].

We propose that the excess of free iron in the CNS could arise from maternal Hb alterations, as in the case of methemoglobinemia. Methemoglobin (MetHb) is defined as a ferric derivative of Hb, that cannot reversibly bind oxygen under physiological partial oxygen pressure [7-9]. In normal conditions the level of MetHb is lower than 1% of the total Hb but it may increase when erythrocytes are affected by a variety of congenital, idiopathic, toxic and environmental factors.

Methemoglobinaemia, that represents a life-threatening complication of exposure to oxidants, is defined by the presence of a greater concentration than the normal physiological level of 1-2% MetHb in erythrocytes. Hemolysis is a well-known consequence of methemoglobinemia. Many of the chemical agents that oxidize hemoglobin to methemoglobin are also capable of inducing erythrocyte injury responsible for hemolysis and consequent release of free iron molecules with toxic properties [48].

Besides, MetHb affects the function of the capillary endothelium of the blood-brain barrier, increasing the apoptotic degeneration and facilitating the passage and the deposition of toxic Fe^3+ ^in the fetal brain [49,50].

Mohorovic et al. [51-53] point out the role of MetHb catabolism and reported that MetHb with prooxidant property is an early biomarker of oxidative stress which places the pregnancy at risk. As source of ferric (Fe3+) form concentrated in various brain regions may impair the health of newborns, children and adults. Pregnant complications, such as anemia, threatened abortion/premature labor and signs of preeclampsia have been observed by Tabacova et al. [54] significantly related to maternal MetHb. Hjell et al. point out that methemoglobinemia can be found also in newborns [55]. These authors observed in a neonatal intensive care unit high concentrations of MetHb among neonates born at 25-30 weeks of gestation and/or with a birth weight < 1000 g.

In most cases MetHb is the result of exposure to oxidant drugs or chemicals, including cigarette smoke. Cigarette smoke is a highly complex mixture of over 7000 chemical compounds distributed in the aqueous, gas and tar phases of the smoke [55]. In the gas phase the smoke comprises high concentrations of oxidants/free radicals (> 10^15 ^molecules per puff) [56,57].

In our study, 11 of the 12 victims of sudden death with iron deposits had a smoker mother. This highly significant correlation supports our view that iron accumulations in the brains of fetuses and newborns could be due to maternal methemoglobinemia, in all likelihood an effect of oxidants in cigarette smoke.

Free radical species may also be due to air pollution. Oxidative stress accompanied by increases of ROS production is, in fact, one of the mechanisms through which airborne pollutants, such as oxidant gases (ozone, nitrogen dioxide, sulphur dioxide) or particulate matter cause adverse effects on human Hb [58,59].

Unfortunately, even if MetHb can be rapidly quantitated by spectrophotometry, in our study data on the maternal Hb typing were not available.

## Conclusions

Our research work will continue with the aim of confirming the involvement of the mother's MetHb level and of iron, as its catabolic product, in causing neuronal damage in victims of sudden fetal death and SIDS.

If this should prove true it will be of fundamental clinical importance to develop preventive treatment strategies that can be applied before irreversible fetal brain damage occurs. Therefore, it is extremely useful to evaluate the plasma MetHb concentration in pregnant women and, in view of the evidence that maternal smoking is one of the main contributors to Hb alterations, to warn women that smoking places their fetus at serious risk of brain abnormalities that could also lead to sudden, apparently unexplained death.

## Competing interests

The authors declare that they have no competing interests.

## Authors' contributions

AML planned the study, analyzed the data and wrote the manuscript with collaborative input and extensive discussion with LucMo and LuiMa. GF and MFC carried out the histochemical study and participated in the evaluation of the results. All Authors read and approved the final manuscript.

## Funding

No financial assistance was received to support the study

## Pre-publication history

The pre-publication history for this paper can be accessed here:

http://www.biomedcentral.com/1471-2431/11/62/prepub
